# Supplementary figures and images for: SCARECROW gene function is required for photosynthetic development in maize
Source: Plant Direct. 2020 Sep 9;4(9):e00264. doi: 10.1002/pld3.264 (PMC7507539; doi:10.1002/pld3.264)

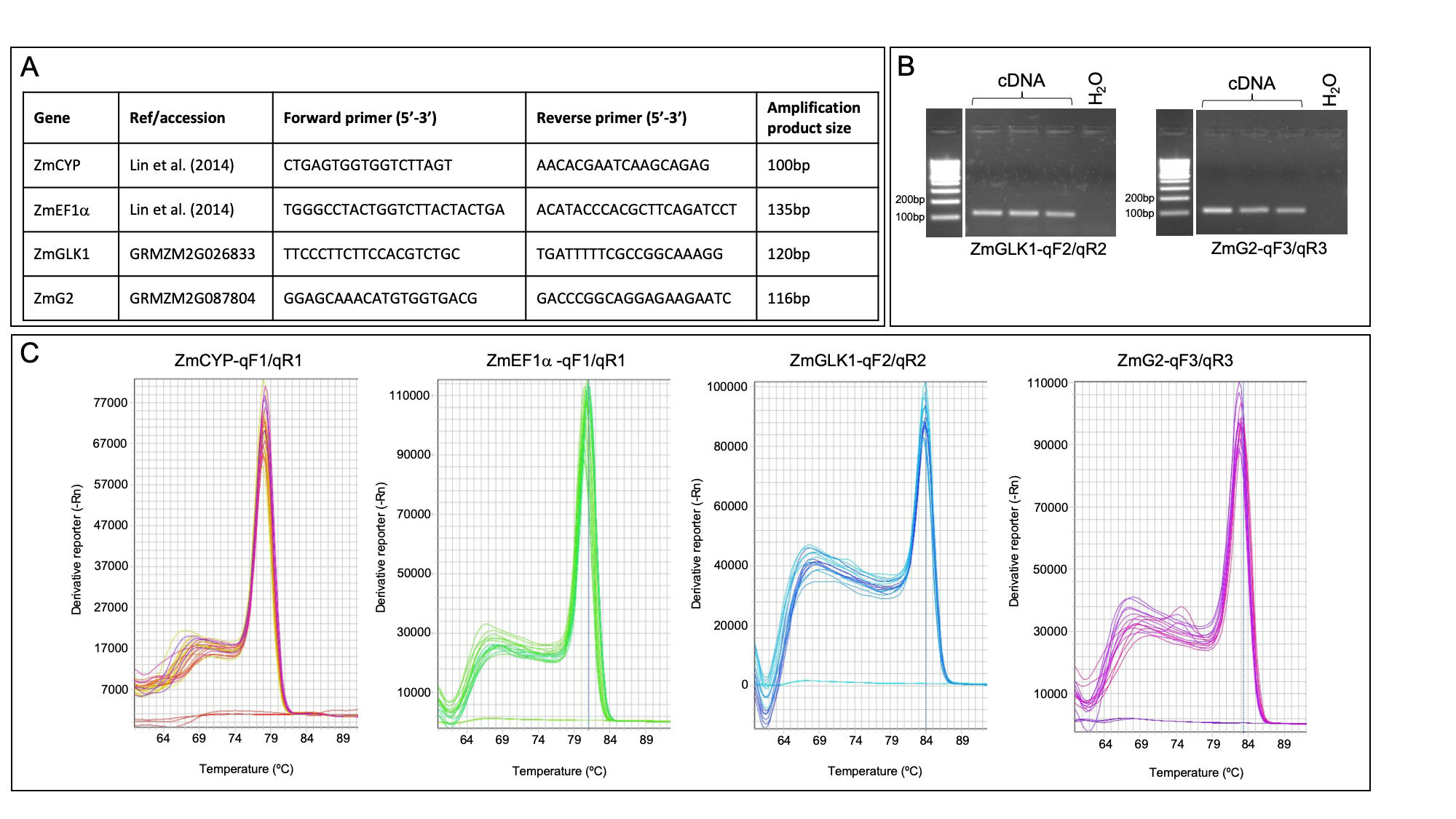

Supplement: Supplementary file 1 — Figure S1 [file PLD3-4-e00264-s001.tiff]

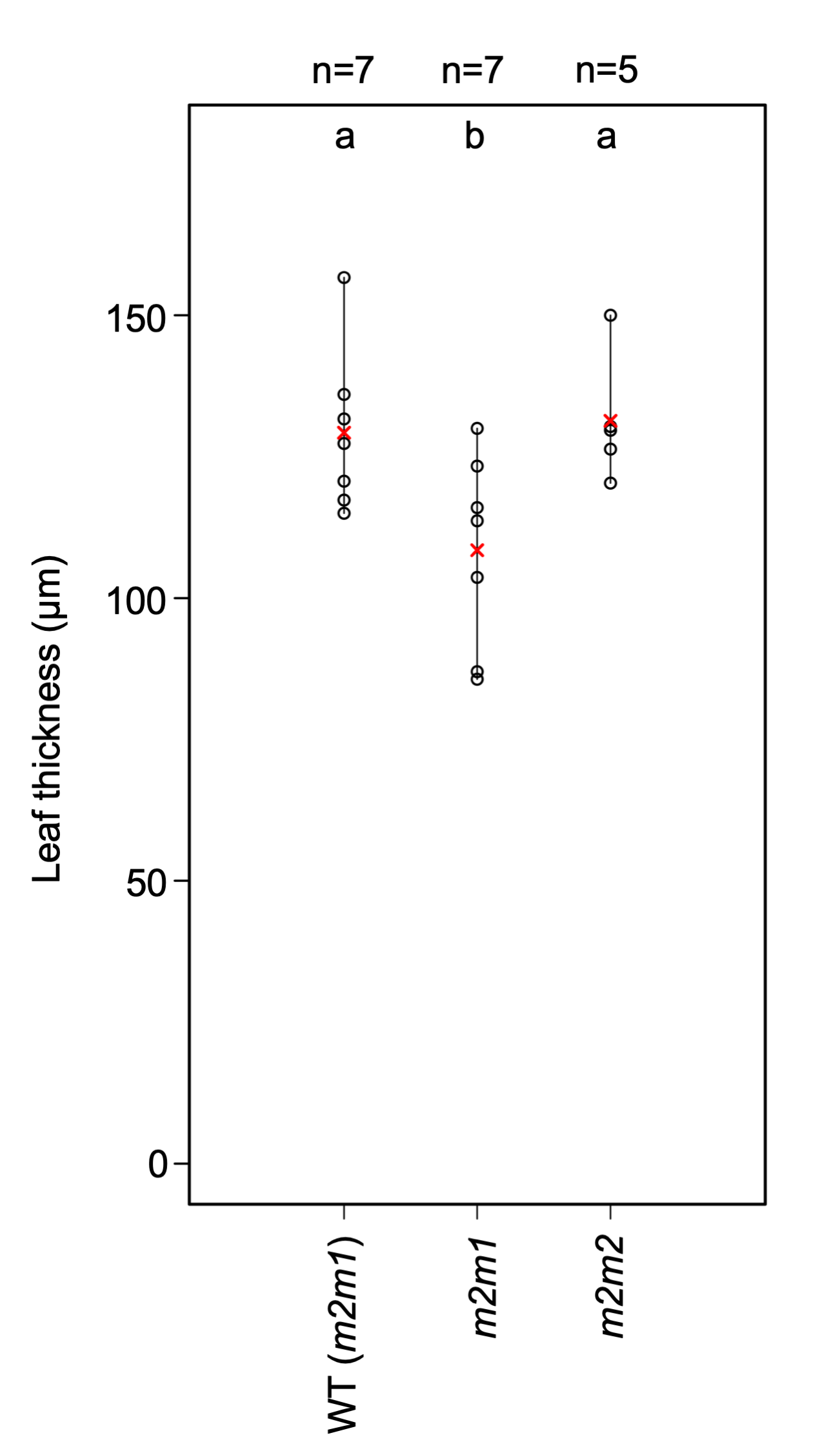

Supplement: Supplementary file 2 — Figure S2 [file PLD3-4-e00264-s002.tiff]

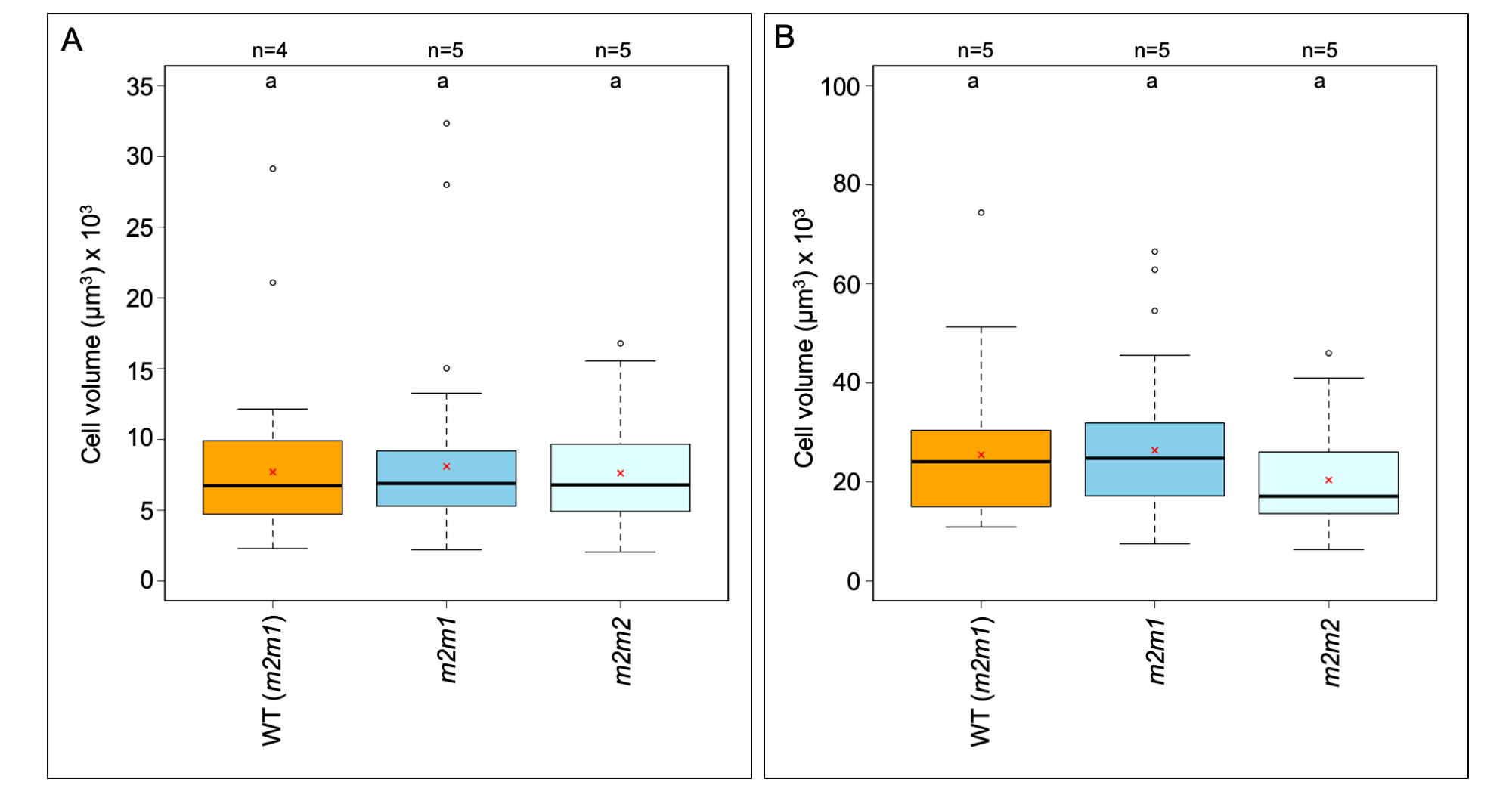

Supplement: Supplementary file 3 — Figure S3 [file PLD3-4-e00264-s003.tiff]

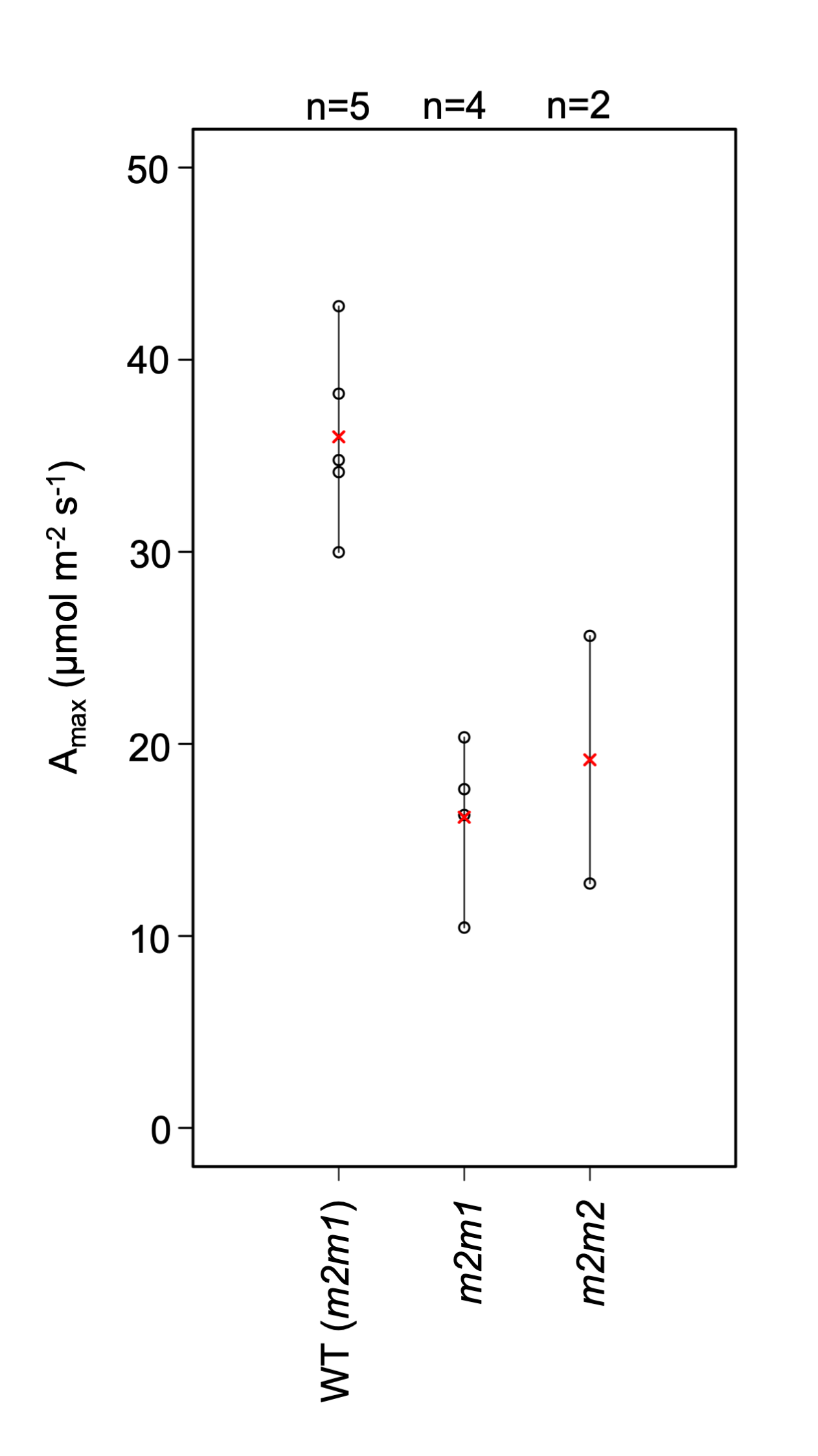

Supplement: Supplementary file 4 — Figure S4 [file PLD3-4-e00264-s004.tiff]

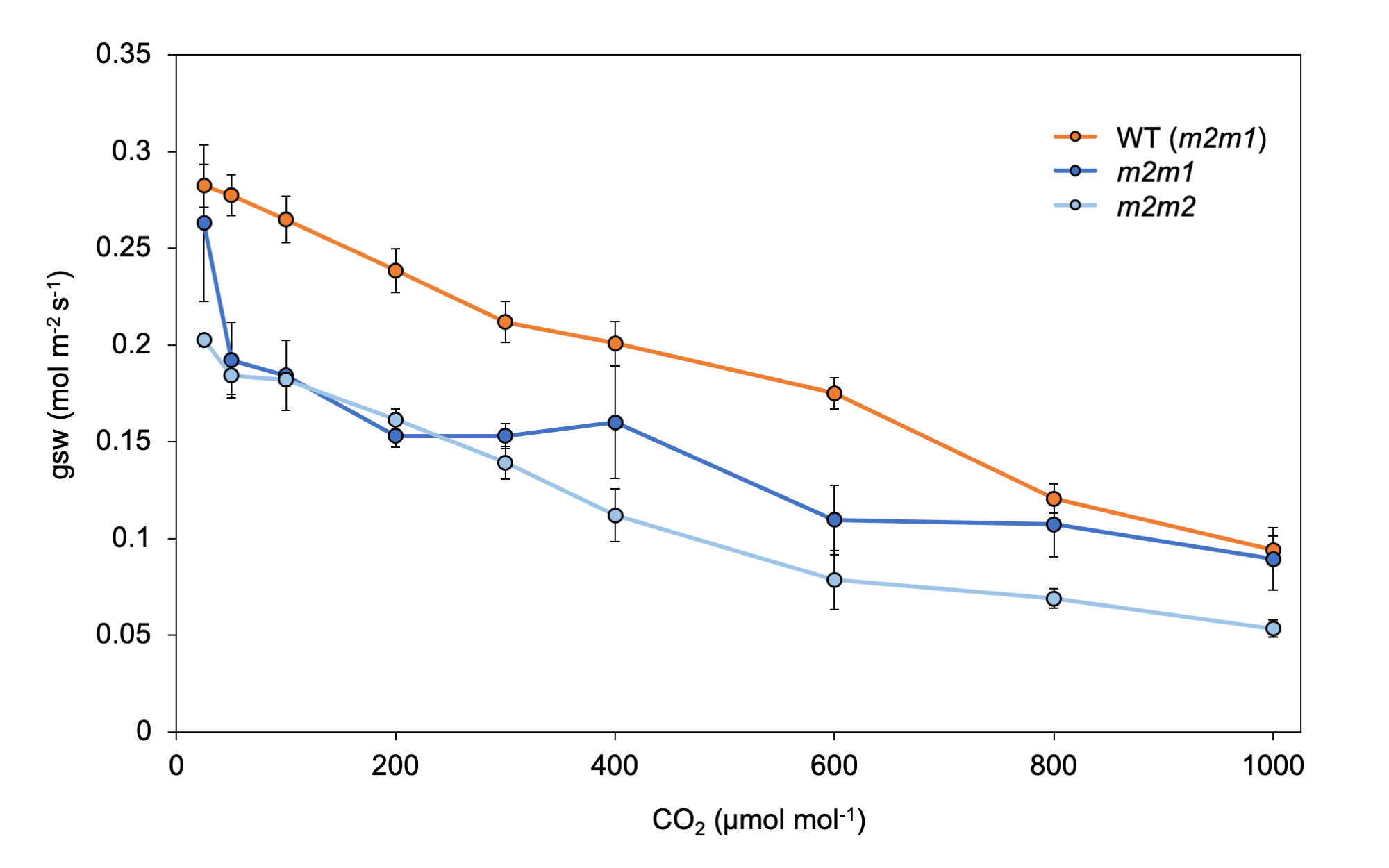

Supplement: Supplementary file 5 — Figure S5 [file PLD3-4-e00264-s005.tiff]
